# All in this together: Multifactorial stress combination promotes cell aggregation in *Chlamydomonas reinhardtii*

**DOI:** 10.1093/plphys/kiaf576

**Published:** 2025-11-09

**Authors:** Nicola Trozzi, Pablo Ignacio Calzadilla

**Affiliations:** Assistant Features Editor, Plant Physiology, American Society of Plant Biologists; The Mechanobiology Laboratory, Department of Plant Molecular Biology, University of Lausanne, CH-1015 Lausanne, Switzerland; Assistant Features Editor, Plant Physiology, American Society of Plant Biologists; Institute for Integrative Biology of the Cell (I2BC), Université Paris-Saclay, CEA, CNRS, Gif-sur-Yvette cedex 91198, France; Department of Earth and Environmental Sciences, Faculty of Science and Engineering, University of Manchester, Manchester M13 9PT, UK

Climate change and increasing anthropogenic pollution pose new challenges to ecosystems and the organisms that inhabit them. The growing intensity of extreme and fluctuating weather conditions raises the number of stressors to which these organisms must respond (Schaeffer et al. 2025). These stressors often occur simultaneously or sequentially, impacting organismal survival and reducing ecosystem services and biodiversity (Zandalinas and Mittler 2022; Schaeffer et al. 2025). A conceptual framework known as the multifactorial stress combination (MFSC) principle has been proposed to address the simultaneous exposure to multiple stresses in different organisms (Zandalinas and Mittler 2022). This principle implies that the combined effect of several stressors imposed simultaneously is greater than the sum of the individual effects. Although MFSC responses have been explored in plants and in soil microbial communities, mainly addressing diversity and respiration changes (Rillig et al. 2019; Zandalinas et al. 2021), how multifactorial stress combinations impact unicellular photosynthetic microorganisms still remains elusive. Identifying the general signaling and metabolic pathways underlying MFSC responses will improve our understanding of how natural and agricultural ecosystems respond to changing climates.

In this issue of *Plant Physiology*, Pascual et al. (2025) investigated the effects of MFCS exposure, including salinity, cadmium, excess light, acidic pH, and the herbicide paraquat (i.e. methylviologen), on the green alga *Chlamynas reinhardtii*. The authors examined how increasing the number of simultaneous stresses affected a wild-type strain and a mutant lacking the respiratory burst oxidase homolog RBOH1 (hereafter *rbo1*), an enzyme involved in the production of reactive oxygen species (ROS) (Fichman et al. 2023). Growth rate, chlorophyll content, and hydrogen peroxide (H₂O₂) accumulation were measured after 24 h of stress exposure. Growth and chlorophyll content declined progressively with the addition of each new stressor but to a similar extent in *rbo1* and wild-type. In contrast, extracellular H₂O₂ levels increased when cells were subjected to more than 2 combined stresses. This accumulation was higher in the wild type than in *rbo1*, consistent with the role of RBOH1 in ROS production (Fichman et al. 2023).

During the phenotypic characterization of *C. reinhardtii*, the authors observed the formation of cell aggregates in response to MFSC. Aggregation occurred even under a single stress exposure but increased progressively with the number of combined stresses. However, while under mild stress combinations aggregation levels were similar between the wild type and *rbo1*, stress treatment aggregation was larger in *rbo1* than in the wild type under the 5-MFSC. The *rbo1* also showed some aggregation even under control conditions. Since the *rbo1* and wild-type strains responded similarly to stress in terms of growth rate and chlorophyll content, differential aggregation could result from contrasting extracellular H₂O₂ levels (Smirnoff and Arnaud 2019). To test this hypothesis, Pascual et al. (2025) examined the effect of exogenous H₂O₂ on cellular aggregation. Application of low H₂O₂ concentrations (0.5 or 1.5 *µ*M) induced similar clustering in *rbo1* and wild-type cells, whereas higher concentrations (3.5 and 5 *µ*M) triggered a stronger aggregation response in *rbo1*. This indicates that *rbo1* cells are more sensitive to external H₂O₂, consistent with altered ROS perception and signaling in the mutant. Together, these findings suggest that H₂O₂ acts as a signaling molecule for cell adhesion (Fig.), consistent with reports describing the involvement of ROS in cell-to-cell communication (Smirnoff and Arnaud 2019).

**Figure.**
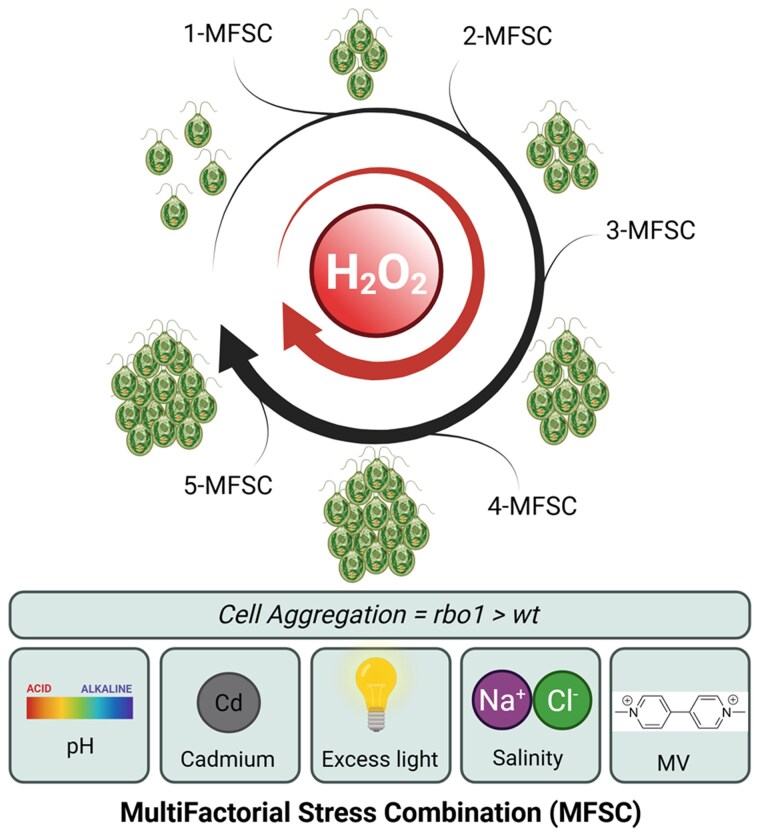
MFSC promotes cell aggregation in *C. reinhardtii*. Under a combination of different abiotic stresses, *C. reinhardtii* cells form multicellular aggregates. Aggregation intensity increases with the number of combined stresses and is highest in *rbo1* mutants compared with wild-type cells. Exogenous hydrogen peroxide (H₂O₂) enhances this response, suggesting that ROS signaling is involved in cell aggregation. MV, Methylviologen. Created with Biorender.com.

Cellular aggregation in unicellular organisms is associated with abiotic stress responses but can also result from cell death (de Carpentier et al. 2022). To test whether MFSC-induced aggregation was linked to cell death, Pascual and colleagues examined the recovery of *rbo1* and wild-type cells after stress exposure. Growth rates and the photosynthetic parameter Fv/Fm, which indicates PSII performance, were monitored during a 72-hour recovery period. Despite the severity of the 4- and 5-factor MFSC treatments, both *rbo1* and wild-type cells regained their growth and photosynthetic efficiency once stresses were removed. This demonstrated that aggregation was not caused by cell death but rather a stress acclimation response.

To further investigate the molecular pathways underlying MFSC responses, the authors performed whole-cell proteomics on *rbo1* and wild-type strains subjected to either single or combined stresses for 24 h. Distinct proteomic profiles were observed for individual stresses, while multifactorial stress treatments revealed shared pathways related to amino acid and pigment metabolism, motility, and cytoskeleton organization. Proteins associated with cilia function, cytoskeletal remodeling, and membrane dynamics increased under high-level MFSC, especially in *rbo1* cells, consistent with enhanced aggregation in this mutant. Antioxidant responses, however, differed between genotypes: glutathione peroxidase 5 (GPX5) increased in wild type but not in *rbo1*, whereas cytochrome c peroxidase accumulated specifically in the mutant. These patterns suggest that the absence of RBOH1 alters ROS perception and scavenging in the mutant, increasing sensitivity to oxidative signals that promote aggregation.

Considering that most of the work on MFSC responses has been conducted in higher plants (Rillig et al. 2019; Zandalinas et al. 2021), Pascual and colleagues searched for common MFCS responses in *Arabidopsis thaliana*, rice (*Oryza sativa*), and *C. reinhardtii*. The authors identified 165 shared components enriched in pathways related to translation, amino acid biosynthesis, nitrogen metabolism, and redox regulation. This overlap indicates that unicellular algae and plants rely on a common molecular framework to withstand complex environmental stress combinations. Yet the emergence of aggregation in *C. reinhardtii* reveals a distinct strategy for single-celled organisms, linking molecular stress responses to collective physical behavior.

In summary, Pascual et al. (2025) shed light on the response of *C. reinhardtii* to MFSC, extending the concept of stress responses from a physiological scale to multicellular organization. Reactive oxygen species, often regarded as damaging byproducts (Smirnoff and Arnaud 2019), emerge here as regulators of a coordinated response to multiple stressors, driving transient multicellularity under environmental pressure. The findings also raise an evolutionary question: if environmental stress favored aggregation in unicellular photosynthetic organisms, similar mechanisms may have contributed to the emergence of stable multicellular lineages (Ratcliff et al. 2013). As ecosystems increasingly experience overlapping global change factors (Zandalinas and Mittler 2022), understanding how unicellular organisms integrate stress signals and form collective structures could reveal both the ecological consequences of anthropogenic activity and the origins of biological cooperation.

## Recent research articles in *Plant Physiology*

- Sinha et al. (2024) studied how MFSC impacted commercial crop cultivars, particularly rice and maize.
- Zagorščak et al. (2025) addressed how single and combined abiotic stress treatments affect potato acclimation responses using a multi-omic perspective.
- Jiang et al. (2024) examined the morphological, physiological, and molecular responses to simultaneous or sequentially imposed stresses in *Arabidopsis thaliana*.
